# Does the preoperative fasting period affect the liver in a distant organ model of renal ischaemia reperfusion?

**DOI:** 10.15537/smj.2023.44.2.20220582

**Published:** 2023-02

**Authors:** Yasemin Akcaalan, Ersin Gurkan Dumlu, Ebru Menekse, Mustafa Cem Yilmaz, Ezgi Erkilic, Betul Ogut, Aylin Sepıcı Dıncel

**Affiliations:** *From the Department of Anesthesia and Reanimation (Akcaalan, Yilmaz, Erkilic), Ankara City Hospital, from the Department of General Surgery (Dumlu, Menekse), Ankara Yıldırım Beyazıt University, from the Department of Pathology (Ogut); and from the Department of Biochemistry (Dıncel), Medicine Faculty, Ankara Gazi University, Ankara, Turkey.*

**Keywords:** ERAS, ischaemia reperfusion, preoperative fasting

## Abstract

**Objectives:**

To experimentally evaluate the effects of preoperative fasting duration on distant organ liver in renal ischaemia-reperfusion (IR) injury.

**Methods:**

This is an experimental study. In the study, 3 groups were formed. In Group A, abdominal laparotomy was carried out after 12 hours of preoperative fasting without any IR damage. In Group B, IR injury was carried out after 12 hours of preoperative fasting, and abdominal laparotomy was carried out, in Group C after 2 hours of fasting after IR injury. Apoptosis, congestion, balloon degeneration, nuclear pleomorphism, and leukocyte infiltration were examined histopathologically and tumor necrosis factor-alpha (TNF-α), interleukin (IL) -1 beta, IL-6, and IL-10 were evaluated biochemically.

**Results:**

A statistically significant difference was determined between the groups in respect of postoperative IL-10 levels (*p*=0.020) with significantly lower levels determined in Group C than in Groups A and B (*p*=0.021). Similar rates of mild nuclear polymorphism were seen with no statistically significant difference determined between the groups (*p*>0.167). A statistically significant difference was determined between the groups in respect of the congestion scores (*p*<0.001), with a lower score in Group C than in Groups A and B, where the scores were similar (*p*<0.001, *p*=0.017).

**Conclusion:**

With this result, it would be correct to say that the short preoperative fasting period has protective effects on the liver tissue.

**R**enal ischaemia reperfusion (IR) damage can occur for many reasons, but usually due to shock, sepsis, organ transplantation, and vascular surgery.^1^ High mortality rates can be seen with this condition linked to distant organ failure such as the heart, lungs, brain, and liver.^1^ The liver and the kidneys are organs that work jointly in the regulation of the homeostatic mechanisms of the body, and the metabolism and excretion of drugs and toxic products.^2^ There are studies in the literature that have defined a close cross-linked relationship between the kidneys and the liver in IR models.^2,3^

The distant liver effect asssociated with renal IR continues to be a subject of research. Although the pathogenesis of distant organ damage is thought to be multifactorial, the formation of reactive oxygen species (ROS) is the mechanism usually held responsible.^4^ The destructive effects of renal IR damage on liver tissue and function have been shown to be formed through reducing antioxidant capacity by triggering oxidative stress and inflammation.^5^ Together with the inflammatory process started by hypoxia and IR damage, several inflammatory molecules start to be released from neutrophils, lymphocytes, macrophages, and primarily endothelial cells. The important inflammatory molecules released are tumour necrosis factor alpha and beta (TNF-α, TNF-β), some interleukins (IL-1β, IL-2, IL-6, IL-11, IL-12, and IL-17), and adhesion molecules.^6^

Preoperative fasting of patients causes several perioperative metabolic changes. Studies have shown that a prolonged fasting period increases insulin resistance, the inflammatory response to surgery, and oxidative stress.^7^ The response of the body to insulin starts to decrease, accompanied by glucogenolysis, gluconeogenesis, and fatty acid oxidisation. At this stage, insulin resistance halts the cellular intake of glucose and the glucogen reserves in the liver starts to empty.^8^ Hyperglycemia is seen in the circulation associated with insulin resistance. Immune, endothelial, and neural cells are affected by this condition, organ damage and infections occur, primarily in the kidneys and liver, and glycolysis is stimulated. Consequently, more free radicals are formed inflammatory markers rise.

Enhanced Recovery After Surgery (ERAS) protocols have gained popularity in recent years. This is an evidence-based, multidisciplinary treatment method encompassing the perioperative period, which aims to minimize organ dysfunction and accelerate patient recovery and discharge.^9^ Studies have shown that reducing the preoperative fasting period, providing sufficient hydration, and the consumption of carbohydrate-rich drinks within this program are safe.^9^

The preoperative administration of carbohydrate also causes a lesser acute inflammatory response. Studies have demonstrated lower C-reactive protein (CRP) and IL-6 levels in patients given preoperative carbohydrates compared to patients with a long preoperative fasting period.^10,11^

In renal IR models in the literature, there is no clear proven information related to the effects of the preoperative fasting period on distant organ liver tissue. The aim of this study was to evaluate the effects of the preoperative fasting period on the liver as a distant organ in an experimental model of renal IR. Tissue pathologies were examined with the biochemical parameters of TNF-α, IL-1β, IL-6, and IL-10, which are markers of increased oxidative stress and the release of free radical oxygen molecules in the liver following IR.

## Methods

Approval for the study was granted by the Animal Research Ethics Committee of Health Sciences University Ankara Training and Research Hospital, Ankara, Turkey (meeting number: 66, decision no.: 666, dated: 26.07.2021). This experimental study was started in 2021 after the approval of the ethics committee and the study was carried out in the Animal Laboratory at Ankara Training and Research Hospital, Ankara, Turkey. The study sample comprised 21 male Wistar albino rats, each aging 8-9 weeks and weighting 250±25 grams. An environment was established before the experiment of room temperature 22-25°C, a 12-hour light/dark cycle (08:00-20:00 light; 20:00-08:00 dark), and humidity in the range of 55-60%. The rats had free access to standard rat food (23% protein, 5% fat, 15% fibre, and 50% carbohydrate) and tap water. The 21 rats were separated into 3 groups of 7. The anaesthesia procedure at the start of the study was applied to all the rats intraperitoneally with 90 mg/kg ketamine (Ketalar Flacon, 50 mg/ml Pfizer, Istanbul, Turkey) and xylazine 10 mg/kg intraperitoneal (Rompun 2% Flacon Bayer, Istanbul, Turkey). A blood sample was obtained from all the rats to determine the preoperative biochemical basal values. Laparotomy was applied to all the rats with a midline incision of approximately 3 cm.

The National Institutes of Health Guiding Principles has been taken as a guide regarding the care and use of animals used in this experimental study.^12^

### Group A

Sham group: following a preoperative 12-hour complete fasting period, abdominal laparotomy was carried out, and liver tissue was removed without the creation of renal ischaemia.

### Group B

Following a preoperative 12-hour complete fasting period, abdominal laparotomy was carried out. The internal organs were retracted and then the left kidney pedicle was clamped. Ischaemia was formed for 20 minutes, then the clamp was opened and a 20-minute period of reperfusion was provided.^13^ Liver tissue was removed following reperfusion.

### Group C

Following a preoperative 2-hour complete fasting period, abdominal laparotomy was carried out. The internal organs were retracted and then the left kidney pedicle was clamped. Ischaemia was formed for 20 minutes, then the clamp was opened and a 20-minute period of reperfusion was provided.^13^ Liver tissue was removed following reperfusion.

### Histopathological evaluation

The liver tissues obtained were placed in dishes containing 10% formaldehyde solution, which had been previously labelled for histopathological examination. After removal of the liver tissue, a second blood sample was obtained from the vena cava inferior of each rat for biochemical examination of the postoperative values. All the rats were then sacrificed with cervical dislocation.

The liver tissues of the rats were fixed in 10% formaldehyde solution for 12-36 hours. The samples were separated into pieces macroscopically and one piece was sampled from each sample. Following routine de-paraffinisation with xylene and graded alcohol solutions, the sections were embedded in paraffin and 4 µm slices were cut. These slices were applied with a hematoxylin and eosin (H&E) staining protocol, and then evaluated under an Olympus BX51 microscope for apoptosis, congestion, balloon degeneration, nuclear pleomorphism, and leukocyte infiltration. Scoring was applied as follows: I) for congestion, 0=none, 1=mild (<50% vascular), and 2=significant (>50% vascular); II) for balloon degeneration, 0=none, 1=mild (occasionally in a few cells), and 2=widespread (at least one cell in a magnification area x100); and III) for nuclear pleomorphism, 0=present and 1=absent.^14^

### Biochemical evaluation

The TNF-α, IL-1β, IL-6, and IL-10 levels in the serum samples were measured with the solid phase sandwich Enzyme-Linked ImmunoSorbent Assay (ELISA) method using the TNF-α ELISA Kit (USCN, Lot: L201013972), IL-1β and IL-10 ELISA Kit (USCN, Lot:L201013957), and the IL-6 ELISA Kit (USCN, Lot: L201013952) according to the manufacturer’s instructions.

### Statistical analysis

Data obtained in this study were analyzed statistically using the Statistical Package for the Social Sciences, version 17.0 (SPSS Inc., Chicago, IL, USA) software. Conformity of continuous variables to normal distribution was assessed with the Shapiro-Wilk test, and homogeneity of variance with the Levene test. Descriptive statistics were stated as median (25th-75th percentiles) for continuous variables and ranked variables were also shown as numbers and percentages. The presence of a significant difference in the biochemical measurements from preoperative to postoperative within the groups was examined with the Wilcoxon signed rank test. The significance of differences between groups in respect of biochemical measurements and histopathological scores were evaluated with the Kruskal-Wallis test. When the results of the Kruskal-Wallis test were found to be significant, the Dunn-Bonferroni test was applied to determine the group causing the difference. Correlations between biochemical measurements and between these and histopathological markers were examined using Spearman’s rank number correlation analysis. In all the potentially multiple comparisons, Bonferroni correction was applied to check type 1 error. A value of *p*<0.05 was accepted as statistically significant.

## Results

### Biochemical results

According to Bonferroni correction, there was no statistically significant difference between the groups in respect to preoperative and postoperative IL-1β, TNF-α, and IL-6 levels (*p*=0.028). The preoperative and postoperative differences were similar for all 3 markers.

The preoperative IL-10 levels were seen to be lower in Group C than in the other 2 groups, but the difference between the groups was not statistically significant (*p*=0.205).

**Figure 1:**
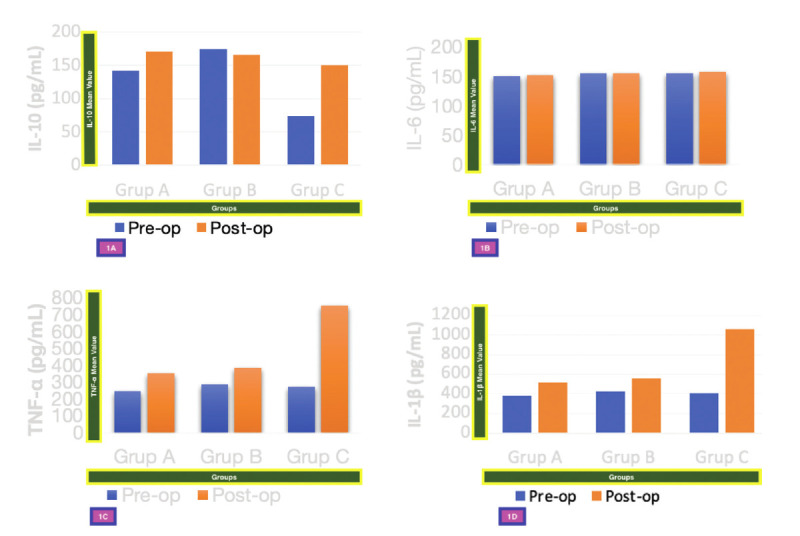
- Variation of cytokine levels in pre-op and post-op blood of rats. A) Statistically significant difference was determined between the groups in respect of postoperative IL-10 levels (*p*=0.020) with significantly lower levels determined in Group C than in Groups A and B (*p*=0.021). B) Within the groups, there was no statistically significant difference between the preoperative and postoperative IL-6 levels according to Bonferroni correction (*p*>0.167). C) Within the groups, there was no statistically significant difference between the preoperative and postoperative TNF-α levels according to Bonferroni correction (*p*>0.167). D) According to Bonferroni correction, there was no statistically significant difference between the groups in respect of preoperative IL-1β levels (*p*=0.028). IL-6: interleukin 6, IL-10: interleukin 10, TNF-α: tumor necrosis factor alpha, Pre-op: preoperative, Post-op: postoperative, IL-1β: interleukin-1 beta

A statistically significant difference was determined between the groups in respect of postoperative IL-10 levels (*p*=0.020) with significantly lower levels determined in Group C than in Groups A and B. Statistically significant *p*-value between groups was in Group A-C (p=0.020) and Group B-C (*p*=0.021, Figure 1).

According to the results obtained, there was a correlation between IL-1β and TNF-α in respect to the preoperative, postoperative, and change from preoperative to postoperative levels. When the IL-10 levels increased postoperatively compared to the preoperative level, the IL-1β and TNF-α levels also increased (r=0.634, *p*=0.002).

### Histopathological results

**Figure 2:**
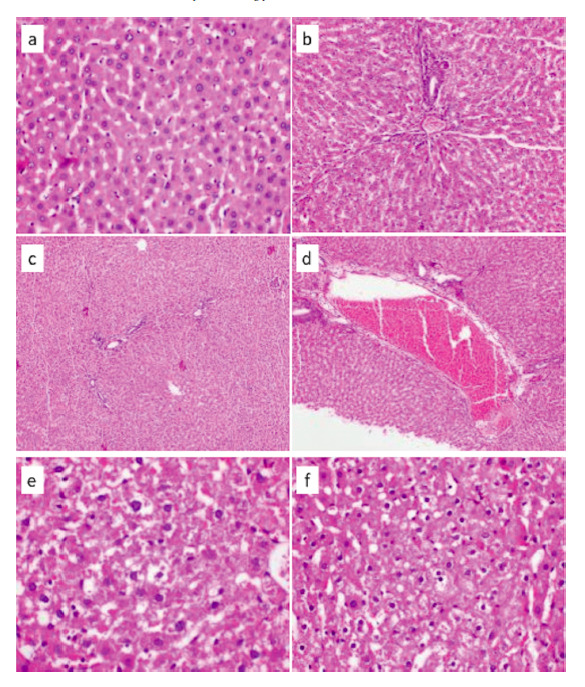
- Histopathological images of liver tissue in rats. a) Normal liver parenchyma (Group C, Rat 1), showing no congestion, nuclear pleomorphism, or ballooning degeneration at high power (200X). b) No parenchymal or portal neutrophil leukocyte infiltration was observed in any liver tissue at high power (40X). c) Liver tissue with congestion score 0 (Group C, Rat 4); and d) at high power (40X). e & f) Congestion score 2 (Group B, Rat 4) at high power (200X). Biopsies with ballooning degeneration and nuclear pleomorphism.

The results of the effects of renal IR were examined with pathological examination of the liver tissues to determine histopathological changes. Accordingly, apoptosis, congestion, leukocyte infiltration, cytoplasmic vacuolar degeneration, and the formation of nuclear polymorphism were examined (Figure 2). Similar rates of mild nuclear polymorphism were seen with no statistically significant difference determined between the groups (*p*>0.167). A statistically significant difference was determined between the groups in respect of the congestion scores (*p*<0.001), with a lower score in Group C than in Groups A and B, where the scores were similar (*p*<0.001, *p*=0.017). No statistically significant difference determined between the groups in respect to vacuolar degeneration (*p*>0.167) although there was a greater number of rats in Group B with significant vacuolar degeneration (Table 1).

**Table 1:** - The scores related to the histopathological markers in the liver tissues of the groups.

Histopathological markers	Group A	Group B	Group C
***Congestion***			
0	-	-	4 (57.1)
1	4 (57.1)	3 (42.9)	1 (14.3)
2	3 (42.9)	4 (57.1)	2 (28.6)
***Vacuolar degeneration***			
0	5 (71.4)	4 (57.1)	5 (71.4)
1	1 (14.3)	-	2 (28.6)
2	1 (14.3)	3 (42.9)	-
***Nuclear polymorphism***			
0	4 (57.1)	4 (57.1)	3 (42.9)
1	3 (42.9)	3 (42.9)	4 (57.1)
2	-	-	-

Values are presented as numbers and precentages (%).

## Discussion

In this study, the effects of renal IR on liver damage were evaluated with biochemical and histopathological parameters in a rat model. Following the 20-minute reperfusion period after ischemia, both IL10 level and liver congestion and vacuolisation were seen to be less in the group with a shorter fasting period due to less inflammation and organ damage.

Hesketh et al’s^15^ 20 minutes slight damage with ischemia, 22 minutes moderate damage with ischemia, and 24 minutes histopathologically proved that there is ischemia and severe tubular necrotic damage. In our study, we used the warm IR model in accordance with the literature, we applied ischemia for 20 minutes to allow mild damage instead of severe necrotic damage in order to compare the effects of a condition on IR damage rather than administering a drug or substance.

The effect on the liver in the early stages of kidney damage after IR is thought to start with an increase in oxidative stress with proinflammatory cytokines, a decrease in anti-oxidants, and the formation of apoptosis. In an experimental study by Golab et al,^1^ it was reported that following IR or bilateral nephrectomy, kidney damage alone and independently of the etiology could cause liver damage. Kelar et al^16^ evaluated the extra-renal effects of acute kidney damage and it was reported that increasing levels of TNF-α and growth factor expressed from hepatocytes had an effect.

Hussein et al^17^ applied renal ischaemia for 30, 45, and 60 minutes followed by a one hour of reperfusion in a rat model, and in contrast to the current study, found the TNF-α levels to be high in all 3 groups. It can be considered that if the reperfusion period in the current study had been longer, different values could have been determined. In addition, in the histopathological evaluation of the study by Hussein et al,^17^ congestion and hydropic degeneration were determined in the 45-minute group and leukocyte infiltration in the 60-minute group. No leukocyte infiltration was determined in any group in the current study, less congestion was observed in Group C, applied with the shorter fasting period, and numerically more vacuolar degeneration was seen in Group B.

In another study by Mehri et al,^6^ 60 minutes of reperfusion was applied after 3 different periods of renal ischaemia of 30, 45, and 60 minutes. Liver functions and the effects on tissue were examined. When the IL-10 and TNF-α levels were evaluated in the liver tissue, the greatest damage was determined in the 60-minute ischaemia group. In our study, we found that the level of IL-10, an anti-inflammatory mediator, was lower in the 2-hour fasting group, due to less inflammation caused by the short fasting period.

Enhanced Recovery After Surgery is defined as the development of multidisciplinary perioperative protocols, including many applications to improve the patient outcomes. The aim of ERAS is to reduce surgical stress. A reduced preoperative fasting period has been proven to have several positive effects for the patient after surgery, such as shorter length of stay in hospital, reduced insulin resistance, and thereby more rapid wound healing and fewer postoperative complications.^8^ However, the subject of preoperative fasting remains a matter of debate. Although nil by mouth from midnight before an operation, reduces the risk of pulmonary aspiration, other benefits are not known.^18^ A long period of fasting causes an increase in insulin resistance and catabolic pathways, and this condition can trigger postoperative complications.

While preoperative fasting can activate endogenous resistance to stress, the preoperative consumption of carbohydrates can improve subjective well-being measurements of the patient, such as thirst, hunger, irritability, and headache associated with preoperative fasting.^19^ In patients with a short fasting period, peripheral glucose uptake and oxidisation in the early period are reduced.^20^

Van Hoorn et al^21^ investigated the effects of IR damage with preoperative nutrition periods and reported that a short fasting period reduced IR damage and protected organ function. In the current study, the effects on liver tissue of IR damage were evaluated with different fasting periods. Better scores were obtained in the histopathological examinations in the group with a short fasting period.

### Study limitations

Examination of a limited number of parameters due to inadequate laboratory conditions.The problem of extrapolating pharmacological data from preclinical to clinical must not be neglected.

In conclusion, in our study, it was observed that short preoperative fasting period in renal IR injury caused less difference in both inflammatory parameters and liver histopathology compared to long fasting period. From these results, it would be correct to say that a short preoperative fasting period has protective effects on liver tissue, as a distant organ.
